# Audiometric evaluation in individuals with mucopolysaccharidosis

**DOI:** 10.6061/clinics/2018/e523

**Published:** 2018-11-23

**Authors:** Marcela Rosana Maia da Silveira, Ana Karina Lima Buriti, Ana Maria Martins, Daniela Gil, Marisa Frasson de Azevedo

**Affiliations:** Fonoaudiologia, Universidade Federal de Sao Paulo (UNIFESP), Sao Paulo, SP, BR

**Keywords:** Mucopolysaccharidosis, Hearing, Hearing Loss, Child

## Abstract

**OBJECTIVES:**

To characterize the audiometric evaluation and acoustic immittance measures in different types of mucopolysaccharidosis.

**METHOD:**

Fifty-three mucopolysaccharidosis patients were evaluated. The classification consisted of type I (Hurler syndrome, Hurler-Scheie and Scheie syndrome), type II (Hunter syndrome), type III (Sanfilippo syndrome), type IV (Morquio syndrome), and type VI (Maroteaux-Lamy syndrome). Immittance audiometry and play or conventional threshold tone audiometry were used to obtain auditory thresholds and were chosen according to the patient's chronological age and ability to understand/respond to the procedure. The findings were analyzed using descriptive statistics and considering the recommendations for research involving human beings contained in Resolution CNE N° 466/2012.

**RESULTS:**

Fifty-one subjects (96.2%) had hearing loss, and the conductive type was the most frequent. Only two (3.8%) patients presented bilateral thresholds within normal limits, one with type IV mucopolysaccharidosis and the other with type VI. There were 11 individuals (20.8%) with mucopolysaccharidosis type I with mixed hearing loss, 9 (16.9%) individuals with type I with conductive hearing loss and 9 (16.9%) with type VI with conductive hearing loss. Mild hearing loss was most common (37.3%), followed by moderately severe hearing loss (36.3%). The type B tympanometric curve (80.4%) was the most frequent.

**CONCLUSIONS:**

Most of the individuals with mucopolysaccharidosis types I, II, III, IV and VI presented mixed or conductive hearing losses of mild to moderately severe degree, type B tympanograms and an absence of contralateral acoustic reflexes.

## INTRODUCTION

Mucopolysaccharidosis (MPS) comprises a group of diseases caused by a deficiency in the lysosomal enzymes involved in mucopolysaccharide metabolism 1,2. As a result, nondegraded mucopolysaccharides accumulate intracellularly and give rise to large cells with cytoplasmic vacuoles and altered cellular function. MPS is a progressive disorder characterized by the involvement of multiple organs, such as the liver, spleen, heart, blood vessels, bone marrow, lymph nodes, osteoarticular system, eyes and ears. The incidence of MPS is approximately 1 in 29,000 live births 3.

MPS are hereditary and therefore permanent metabolic diseases caused by inborn errors of metabolism (IEM) that lead to altered functioning of certain enzymes 4. Therefore, the classification of MPS is based on the specific enzyme deficiency. The known MPS types are MPS type I per deficient enzyme (α-L-iduronidase); MPS type II per deficient enzyme (iduronate sulfatase); MPS type III per deficient enzyme (heparan-N-sulfatase, N-acetyl-α-D-glucosaminidase; acetyl CoA:α-glucosaminidase; acetyltransferase; N-acetylglucosamine-6-sulfatase); MPS type IV per deficient enzyme (N-acetylgalactosamine-6-sulfatase; β-galactosidase); MPS type VI per deficient enzyme (arylsulfatase-β); and MPS type VII per deficient enzyme (β-glucoronidase).

Clinical manifestations of MPS vary according to the enzyme that is missing in the affected individual. The most common clinical symptoms are facial dysmorphism, communicating hydrocephalus, hearing impairment, neuropsychomotor developmental delay, obstructive airway disease, skeletal abnormalities, heart abnormalities and hepatomegaly 2,5,6.

The hearing loss due to MPS may be conductive, sensorineural or mixed. A study 7 indicated that hearing loss is common in all six types of MPS, with mixed hearing loss being more frequent due to poor middle ear ventilation caused by obstruction of the auditory tube and the presence of thick secretions in the middle ear.

Authors 8 have stated that the auditory impairment present in MPS patients may be due to middle ear infection, ossicle deformity and inner ear (cochlea) abnormalities and alterations in the auditory nerve.

One study 9 described the auditory profile of patients with different types of MPS patients and found that conductive hearing loss was the most prevalent type of hearing loss (63.6%), particularly due to the history of repeated otitis. Another study 10 of children with MPS type IV also suggested that otitis was the most common cause of conductive hearing loss.

It is important to note that the appropriate treatment of MPS described in the literature is enzyme replacement therapy. One author 11 concluded that while enzyme replacement therapy in patients with MPS type I facilitates the control of ear, nose and throat infections and rhinorrhea and improves respiratory quality and sleep, it does not improve other parameters (auditory threshold, tympanometric curves, sleep disorders, hypertrophy of the palatine and pharyngeal tonsils and macroglossia).

It is therefore important to diagnose auditory alterations in MPS patients in all age groups, especially during early childhood, because otological infections can cause irreversible damage to hearing, delaying the child's linguistic development.

Although different studies have noted the auditory manifestations of MPS, few have evaluated patients with all types of the disease. Studies most frequently involve patients with a single type of the disease or are case reports.

Thus, the present study aimed to characterize the audiometric profiles and acoustic immittance measurements of patients with different types of MPS.

## METHOD

This is a descriptive, cross-sectional study conducted at the audiology medical clinic of the hearing disorders program of Universidade Federal de São Paulo- UNIFESP, Departamento de Fonoaudiologia.

Sixty individuals were selected, but seven were excluded from the sample because they could not complete the audiometric test battery. Therefore, the final sample consisted of 53 individuals aged 2 to 23 years (21 females and 32 males) who met the following inclusion criteria: diagnosed with mucopolysaccharidosis of any type in the Reference Center in Inborn Errors of Metabolism (CREIM) of the UNIFESP; completion of audiometric tests.

To examine the association between audiological results and MPS type, the sample was distributed into five groups according to MPS classification 4: type I (MPS I: Hurler, Hurler-Scheie and Scheie syndromes); Type II (MPS II: Hunter syndrome); Type III (Sanfilippo syndrome); Type IV (MPS IV: Morquio syndrome); Type VI (MPS VI: Maroteaux-Lamy syndrome).

Data collection started only after the project received approval from the Research Ethics Committee (REC) of the Universidade Federal de São Paulo (UNIFESP) under protocol n° 02111/08. The adults included in the sample and the legal guardians of the children signed the informed consent form, authorizing participation in the study.

Data collection procedures included anamnesis, otoscopy, conditioned or conventional tonal threshold audiometry, and immittance audiometry. The equipment used to perform these procedures were a Missuri^®^ brand otoscope; pediatric audiometer, PA 2 model, and acoustic immittance meter, AZ7 model, both by Interacoustics; an audiometer, model MA-41, MAICO brand, calibrated according to ANSI 1996 standards; TDH-39 headphones; and MX-41 cushions.

The procedure used to obtain auditory thresholds was chosen based on the chronological age of the child and his/her ability to understand/respond to the procedures. When it was not possible to perform the appropriate procedure for the patient's age, we performed the procedure corresponding to the next youngest age and progressed until the results could be obtained safely.

The following procedures were used:

- Visual reinforcement audiometry (VRA): VRA was performed by means of stimulation-response-visual reinforcement for children aged 6 months to 2 years, as proposed by the authors of previous studies 12,13 and recommended by Azevedo 14. In this study, VRA was performed with pure tones modulated at frequencies (*warble*) from 500 to 4000 Hz at intensities of 80, 60, 40 and 20 dB NA, both in these orders.
- Conditional tonal play audiometry: auditory thresholds for frequencies from 500 to 4000 Hz by air conduction for children aged 2 years to 6 years and 11 months.
- Conventional tonal audiometry: measurement of auditory thresholds for the frequencies from 250 to 8000 Hz by air conduction for individuals aged 7 years and older, and auditory thresholds for the frequencies from 500 Hz to 4000 Hz by bone conduction when the air conduction thresholds were altered at intensities above 15 dB NA in children up to 11 years and 11 months and above 25 dB NA in individuals over 12 years of age.
- The results were classified as normal or altered according to standards mentioned in the literature 15 for pure tone audiometry and according to the type of hearing loss, which could be conductive, sensorineural or mixed. To classify the degree of hearing loss, the Northern and Downs 16 criteria were used for children under 12 years of age, and the classification proposed by Silman and Silvermann was used for individuals over 12 years of age.
- Acoustic immittance measurements: compliance, volume of the external acoustic meatus and the threshold of the acoustic reflex with contralateral stimulation were verified. Tympanometric results were analyzed according to the classification proposed by Jerger 17. Acoustic reflexes were considered normal if they were present between 70-90 dB NS for sound frequencies of 500, 1000, 2000 and 4000 Hz 18-20.

Absolute distributions and univariate and bivariate percentages were obtained for descriptive analysis, and the Kruskal-Wallis test was used for inferential statistics. The margin of error adopted in statistical test decisions was 5%.

## RESULTS

Fifty-three MPS patients diagnosed at the UNIFESP reference center were evaluated. Twenty-one patients were diagnosed with MPS type I, 12 with type II, one with type III, 4 with type IV and 15 with type VI. The ages of the individuals ranged from 2 to 23 years, with a mean of 12.5 years. Males comprised slightly more than half of the subjects (60.4%).

The procedure chosen for the audiological evaluation varied according to the age of the individual. Table 1 shows the type of evaluation used for each MPS type.

Of the 53 individuals evaluated, 51 (96.2%) had hearing loss, and conductive hearing loss was the most frequent type. Twenty-five individuals (47.2%) had bilateral hearing loss, and 5 (9.4%) had unilateral conductive hearing loss; 19 individuals had bilateral mixed hearing loss (35.8%), and 5 (9.4%) had unilateral mixed hearing loss; one individual (1.9%) had bilateral sensorineural hearing loss, and 2 (3.8%) unilateral sensorineural hearing loss, one of whom presented with conductive hearing loss in the other ear and the other of whom presented with mixed hearing loss in the other ear. Only two (3.8%) participants presented thresholds within normal limits: one had MPS type IV, and the other had type VI.

Regarding the most common hearing loss profiles associated with disease type, 11 individuals (20.8%) had MPS type I with mixed hearing loss, followed by 9 (16.9%) who had MPS type I with conductive hearing loss and 9 (16.9%) who had MPS type VI with conductive loss (Table 2).

Table 3 considers the number of ears with any type of hearing loss (102 ears).

Concerning tonal audiometry alterations (51 children - 102 ears), there was a high prevalence of mild hearing loss (37.3%), followed by moderate-severe hearing loss (36.3%). Regarding the type of hearing loss, there was a predominance of the conductive type (53.9%), followed by mixed hearing loss (42.2%).

Regarding the individuals with hearing loss, acoustic immittance measures were analyzed for only 90 ears because in 12 out of the 102 altered ears (11.7%), it was not possible to perform the examination (in one ear due to the presence of a ventilation tube and in 11 ears because the individual refused to undergo the procedure). A Type B tympanometric curve was most frequent (76.5%). A type A curve was observed in four ears with sensorineural hearing loss and in four ears with thresholds within the normal range.

Regarding the relationship between the audiometric tests and the type of MPS, a significant association was found in the variable "audiometric tests results". A total of 63.3% of the ears of patients with MPS type VI had conductive hearing loss. However, the one participant with MPS type III had sensorineural hearing loss in both ears (100%) (Table 4).

**Figure 1:**
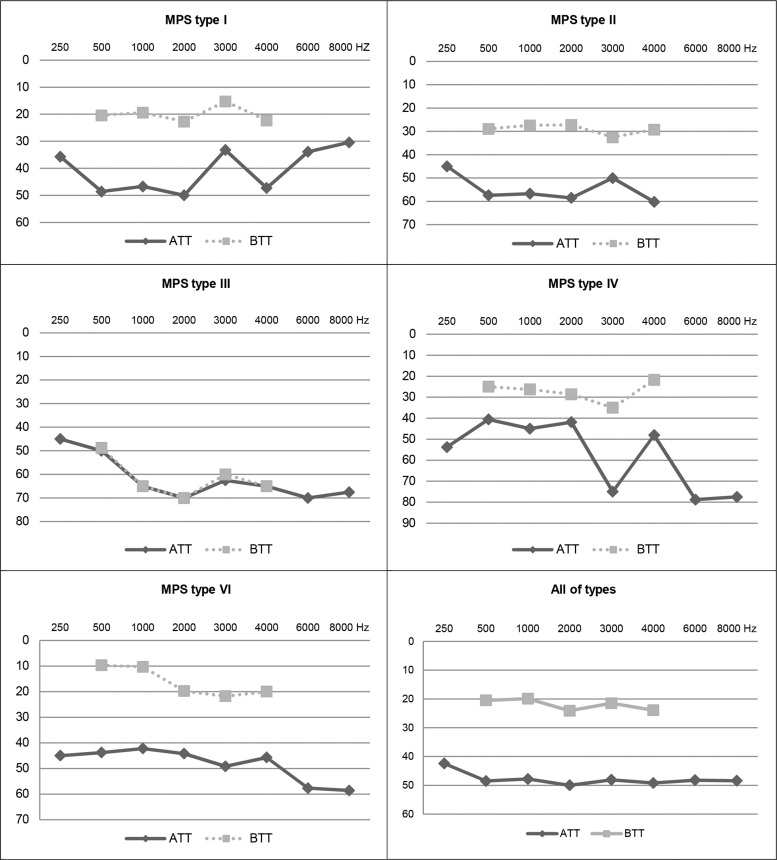
Representation of the audiograms of mean transmission thresholds by frequency (total sample) for each type of MPS. **Legend:** Air transmission threshold (ATT); Bone transmission threshold (BTT); mucopolysaccharidosis (MPS).

Figure 1 shows the mean air and bone conduction thresholds per frequency for each type of MPS and the overall mean of the sample using descriptive analysis.

Individuals with MPS type I had better thresholds at 3000 Hz than those with type IV. Individuals with MPS type I had better auditory thresholds at 6000 Hz and 8000 Hz compared with the other types. The worst auditory thresholds were obtained by individuals with MPS type III compared with types I and VI (Figure 1).

## DISCUSSION

The incidence of MPS has been described in the international literature as 1:29,000 live births 3. The incidence in Brazil is not known, but it has been considered that the population selected for this study is large compared with other studies 21,22 that included a much smaller number of cases or reported isolated cases. Hearing loss was observed in 96.6% of the population evaluated in the present study.

Regarding the procedures used, conventional tonal audiometry was most often performed in patients with MPS type IV and type VI. All the patients with MPS type IV and 75% of the patients with MPS type VI underwent threshold tonal audiometry, which is the indicated procedure for their age (Table 1).

This result was expected because patients with MPS types IV and VI generally present normal intelligence 8. On the other hand, to evaluate MPS type II patients, visual reinforcement audiometry had to be used. Visual reinforcement audiometry is indicated for children aged 6 to 18 months, much younger than the age of the evaluated population, thus indicating the participants' developmental or cognitive impairment. MPS type II individuals experience severe degeneration of the central nervous system, developmental delay, hearing loss, hyperactivity and aggressive behavior 8, which justify the use of procedures more commonly used with younger children.

In the present study, the evaluation of the audiometric results showed no statistically significant difference in auditory thresholds between the ears, indicating bilateral and symmetrical hearing loss in the majority of the patients (Tables 2 and 3).

One individual with MPS type IV and another with MPS type VI had auditory thresholds within normal patterns bilaterally and type A tympanometric curves, revealing good mobility of the ossicular-tympanic system. The low incidence of individuals with normal auditory thresholds and without involvement of the ossicular-tympanic system agrees with the literature 21,23.

Of the total number of ears, 102 presented hearing loss, most of them (96%) with conductive impairment (Table 2) in the form of either conductive or mixed hearing loss, corroborating a study that observed a high prevalence of hearing loss (85%) of different types and degrees of severity in 39 patients with MPS who underwent threshold tonal audiometry (TTA) 23.

Authors 8 studied 21 patients with MPS of different types and observed that all presented auditory alterations, with 18 (85.7%) presenting conductive impairment. Another study 9 reported that 63.6% of individuals with MPS had conductive hearing loss and a history of repeated otitis, corroborating the data of the present study.

In the present study, bilateral mixed hearing loss was observed in 38 ears (35.8% of the sample). Five patients (five ears) presented unilateral mixed hearing loss (4.7% of the sample) accompanied by another type of hearing loss in the other ear. Authors 24 evaluated 45 individuals diagnosed with MPS and found three patients with mixed hearing loss (6.6%), a much lower prevalence than was observed in the present study (Table 2).

Only four ears (3.9%) presented sensorineural hearing loss; one patient had bilateral sensorineural hearing loss, and two patients had unilateral sensorineural hearing loss (Table 3). These data are corroborated by other studies. Authors 8 observed sensorineural loss in 14.3% of MPS patients. A study 9 with MPS patients diagnosed two (18.1%) patients with sensorineural hearing loss.

Table 3 shows that the type B tympanometric curve was the most frequently observed (76.5%), followed by the C curve (7.8%). These data are consistent with a study 21 that observed a higher occurrence of type B tympanograms (77.77%), followed by type C tympanograms (16.66%) and type A tympanograms (5.55%).

An empirical analysis of MPS patients 25 revealed an accumulation of glycosaminoglycans in the neurovascular structures of the inner ear with destruction of Corti's organ, the spiral ganglion and the vascular stria, justifying the occurrence of sensorineural and mixed hearing loss in MPS disease.

An older study 26 also described the histological pathology of the temporal bones of patients with MPS type IV and confirmed the presence of mucopolysaccharide deposits in the middle ear and vascular stria. The authors concluded that sensorineural or mixed hearing loss in children older than 8 years with MPS type IV was due to the deposition of excessive mucopolysaccharides in the cochlear region. In contrast, hearing loss in children aged younger than 8 years with MPS type IV was due to ossicular chain malformations.

In the present study, sensorineural hearing loss occurred only in patients with MPS types I, II and III and was most predominant in type III, which presented the worst auditory bone conduction thresholds. It is noteworthy that only one MPS type III patient was evaluated and diagnosed with bilateral sensorineural loss. Two other ears (in MPS type I and type II patients) that presented sensorineural hearing loss had mixed and conductive loss in the other ear (Table 4). Regarding the degree of loss, the moderately severe form was the most common in individuals with MPS type I (40.5%), followed by mild loss (35.7%) in MPS type I and type VI (36.7%).

A study 27 analyzed 83 patients with MPS type II and previous and sequential hearing loss diagnoses. Seventy patients (94%) were found to have some type and degree of hearing loss. The study concluded that the auditory threshold increased by approximately 1 dB per year, and this change was statistically significant at all frequencies. It is noteworthy that bone conduction thresholds increased with age, indicating that progression of the sensorineural component is part of the natural history of MPS II.

Figure 1 shows the mean audiograms of the individuals in this study by type of MPS. MPS types II and IV showed mean thresholds classified as mixed hearing loss, and only MPS III had characteristics of sensorineural hearing loss. MPS types I and VI presented characteristics of conductive hearing loss. The overall mean thresholds (all types) for all frequencies also indicate a profile of conductive hearing loss. Thus, the high prevalence of conductive hearing loss in individuals with MPS is remarkable.

It is important to emphasize that otological evaluations of MPS patients should be performed as early as possible to avoid middle ear conditions that interfere with the quality of life and language development of young children. Manifestations of otitis media have been highlighted in several studies with patients with MPS, and a higher prevalence of conductive hearing loss has been reported 28, corroborating the findings of the present study.

One author 29 reported two cases of children between 2 and 5 years old with MPS type II (Hunter syndrome), highlighting the risk of middle ear effusion and conductive hearing loss. Otological treatment with placement of ventilation tubes in the middle ear was necessary, and this treatment improved the severe to profound hearing loss by approximately 40 dB (HL), resulting in moderate hearing loss. The author noted that middle ear alterations may mask coexisting sensorineural hearing loss.

Another procedure that improved auditory thresholds was described by authors 30 who studied 27 patients diagnosed with MPS types I, II and III (mean age 19 months) who presented sensorineural hearing loss before stem cell transplantation. After transplantation, there was a statistically significant improvement in the auditory thresholds of 67% of the patients younger than 26 months.

This study described the presence of upper airway infection concomitant with otitis media in patients with MPS types I, II and IV. Hearing loss was reported as a symptom by 45% of the patients studied. There was a high frequency of tympanic membrane retraction (33%). As a result of the data findings and the possibility of irreversible auditory impairments, this study suggests early hearing diagnosis by audiometry and the use of brainstem auditory evoked potentials and otoacoustic emissions for follow-up and intervention in this population 22.

Authors 31 have reported that auditory (conductive, sensorineural or mixed) changes in children with MPS may contribute to late language acquisition, communication difficulties with peers, behavioral problems and the need for special education, in addition to the use of conventional hearing aids and/or cochlear implants.

Therefore, there is the need for early otological intervention by a multidisciplinary team soon after the diagnosis of MPS because studies have reported strong evidence of otitis media episodes, which may lead to auditory changes. Basic hearing assessment (tonal and vocal audiometry and immittance measurement) and periodical follow-up must be performed because of the known progression of hearing loss in this population and the recurrence of middle ear conditions.

The results obtained the present study show the importance of audiological evaluations for children with MPS when the disease is diagnosed in the first years of life to minimize communication difficulties and delayed language development due to sensory hearing loss caused by the high rate of otological infections or by decreased auditory sensitivity. Multidisciplinary intervention can make the diagnosis as early as possible and initiate the most appropriate treatment, thus promoting a better quality of life for affected individuals.

Most individuals with MPS types I, II, III, IV and VI present mixed or conductive hearing loss of mild to moderately severe degree, type B tympanograms, and an absence of contralateral acoustic reflexes.

## AUTHOR CONTRIBUTIONS

Silveira MR was the main researcher, responsible for the elaboration of the research project, preparation of the schedule, literature survey, data collection and analysis. Buriti AK was responsible for the manuscript writing, revision of the text, data analysis, updating the references, final formatting, submission of the manuscript. Martins AM was responsible for the sample selection and patient referral. Gil D was the co-supervisor, responsible for the elaboration of the research project, correction of the data analysis, correction of the final version of the manuscript. Azevedo MF was the supervisor, responsible for the elaboration of the research project, preparation of the schedule, correction of the manuscript and approval of the final version of the manuscript.

## Figures and Tables

**Table 1:** Occurrence of procedures per type of MPS (N=53).

MPS type	TTA		Play audiometry		VRA	
	N	%	N	%	N	%
Type I	10	18.9	2	3.8	9	16.9
Type II	3	5.7	2	3.8	7	13.2
Type III	1	1.9	0	0.0	0	0.0
Type IV	4	7.5	0	0.0	0	0.0
Type VI	12	22.6	0	0.0	3	5.7
Total	30	56.6	4	7.6	19	35.8

Legend: TTA: threshold tonal audiometry, VRA: visual reinforcement audiometry.

**Table 2:** Results of the audiometric evaluation per type of MPS (N=53).

MPS type	Normal		Conductive		Mixed		Sensorineural	
	Bil	Uni	Bil	Uni	Bil	Uni	Bil	Uni
Type I	0	0	9	1	11	0	0	1
Type II	0	0	6	2	3	3	0	1
Type III	0	0	0	0	0	0	1	0
Type IV	1	0	1	1	1	1	0	0
Type VI	1	0	9	1	4	1	0	0
Total*	2	0	25	5	19	5	1	2

**Legend:** Bil = bilateral; Uni = unilateral. * Totals vary due to repeated evaluation of individuals with unilateral hearing loss**.**

**Table 3:** Characterization of altered tonal audiometry and acoustic immittance results per ear (N=102).

Variable	N	%
Type of hearing loss		
Conductive	55	53.9
Sensorineural	4	3.9
Mixed	43	42.2
Degree of hearing loss		
Mild	38	37.3
Moderate	18	17.6
Moderately severe	37	36.3
Severe	9	8.8%
Tympanometric curve		
Type A	4	3.9
Type B	78	76.5
Type C	8	7.8
Not measured	12	11.8
Total	102	100.0

**Table 4:** Characterization of audiometric evaluation results (type and degree of hearing loss) per MPS type in the study group (n=106 ears).

	MPS type												
Variable	Type I		Type II		Type III		Type IV		Type VI		Total group		*p*-value
	%	N	%	N	%	N	%	N	%	N	%	N	%
Audiometric evaluation													
Normal	0	0.0	0	0.0	0	0.0	2	25.0	2	6.7	4	3.8	*p*=0.0743
Conductive	19	45.2	14	58.3	0	0.0	3	37.5	19	63.3	55	51.9	
Sensorineural	1	2.4	1	4.2	2	100.0	0	0.0	0	0.0	4	3.8	
Mixed	22	52.4	9	37.5	0	0.0	3	37.5	9	30.0	43	40.5	
Degree of hearing loss													
Normal	0	0.0	0	0.0	0	0.0	2	25.0	2	6.7	4	3.8	
Mild	15	35.7	9	37.5	0	0.0	3	37.5	11	36.7	38	35.8	
Moderate	8	19.0	2	8.4	0	0.0	0	0.0	8	26.6	18	16.9	
Moderately severe	17	40.5	8	33.3	2	100.0	1	12.5	9	30.0	37	35	*p*=0.3183
Severe	2	4.8	5	20.8	0	0.0	2	25.0	0	0.0	9	8.5	
Total	42	100.0	24	100.0	2	100.0	8	100.0	30	100.0	106	100.0	

* Significant values (*p*<0.05) - Kruskal-Wallis test.
